# Supplementary material for: Combining Electrostimulation with Impedance Sensing to Promote and Track Osteogenesis within a Titanium Implant
Source: Biomedicines. 2023 Feb 24;11(3):697. doi: 10.3390/biomedicines11030697 (PMC10045247; doi:10.3390/biomedicines11030697)
Supplement: Supplementary file 1 [file biomedicines-11-00697-s001.zip › biomedicines-2115623 -supplementary.pdf]

Supplementary Material

Combining Electrostimulation with Impedance Sensing to Promote and Track Osteogenesis within a Titanium Implant

Nadja Engel, Michael Dau, Vivien Engel, Denise Franz, Fabian Klemmstein, Christiane Thanisch, Jürgen F. Kolb, Marcus Frank, Armin Springer, Rüdiger Köhling, Rainer Bader, Bernhard Frerich, Nadine Wiesmann, Diana Heimes and Peer W. Kämmerer

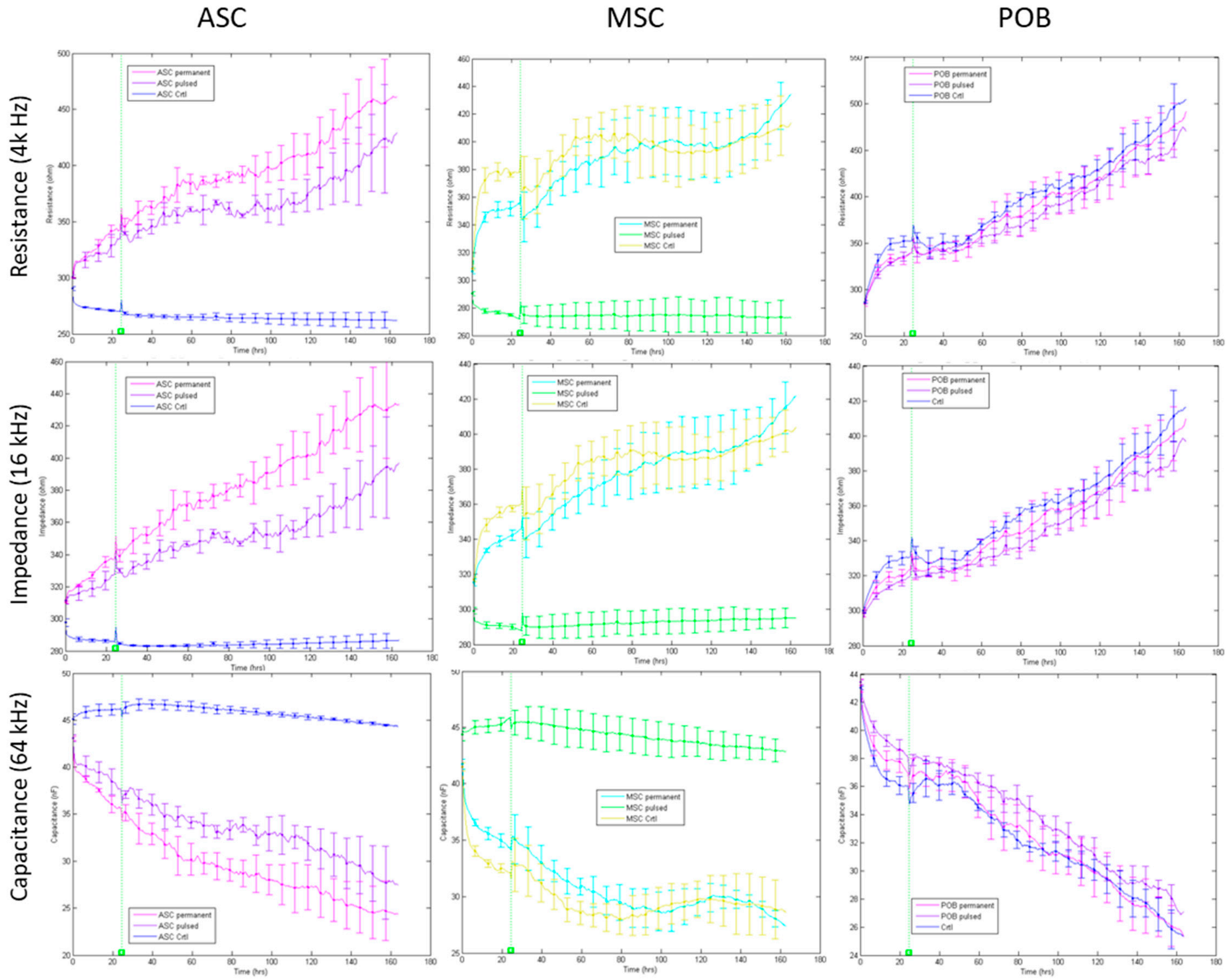

Figure S1. Impedance measurements after 3 days of electrical stimulation. Resistance at 4 kHz, impedance at 16 kHz, capacitance 64 kHz was measured for 7 days in non-stimulated, pulsatile and continuous electrical stimulated ASCs, MSCs and POBs. At t = 0h cells were seeded on the chips. Medium changes occurred every 2 days. mean ± SD, n =3.

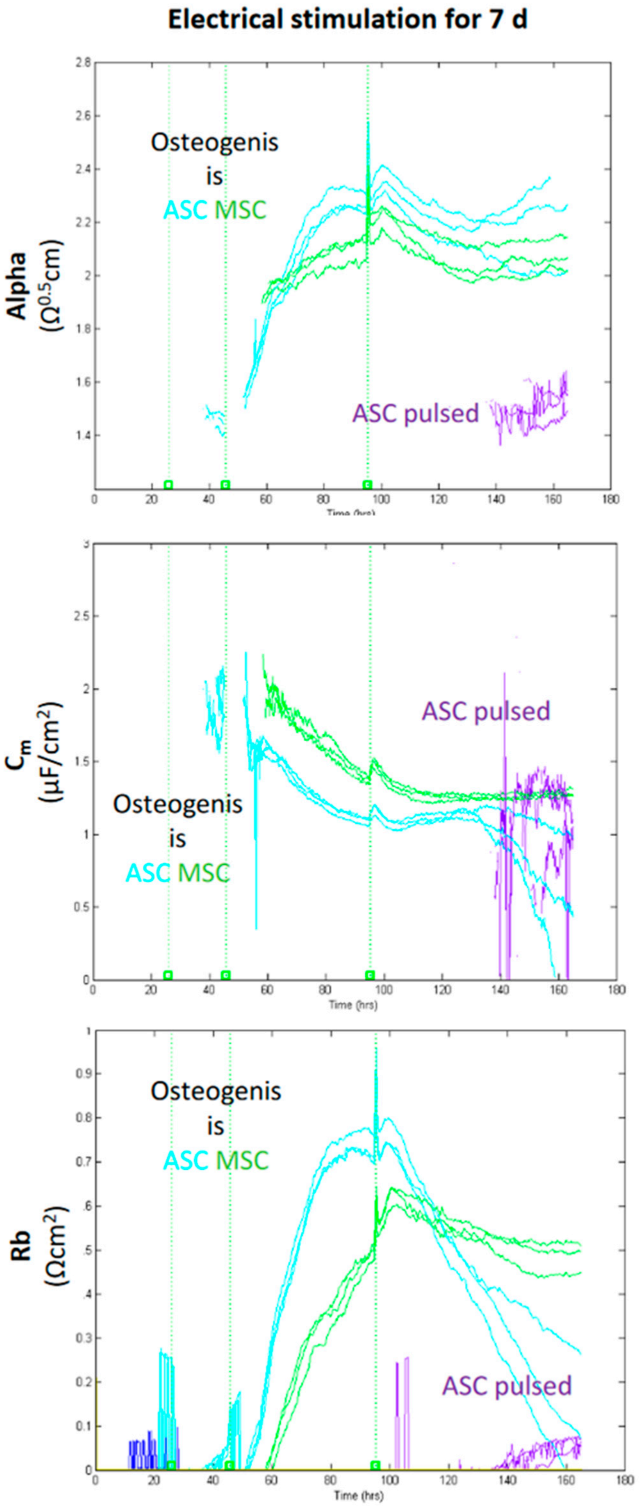

Figure S2. Calculation of resistance (Rb) and cell membrane capacitance (Cm) in osteogen and electrical stimulated stem cells. For the chemical osteogenic stimulated ASCs and MSCs as well as for the pulsatile stimulated ASCs an decrease of Cm was detected.

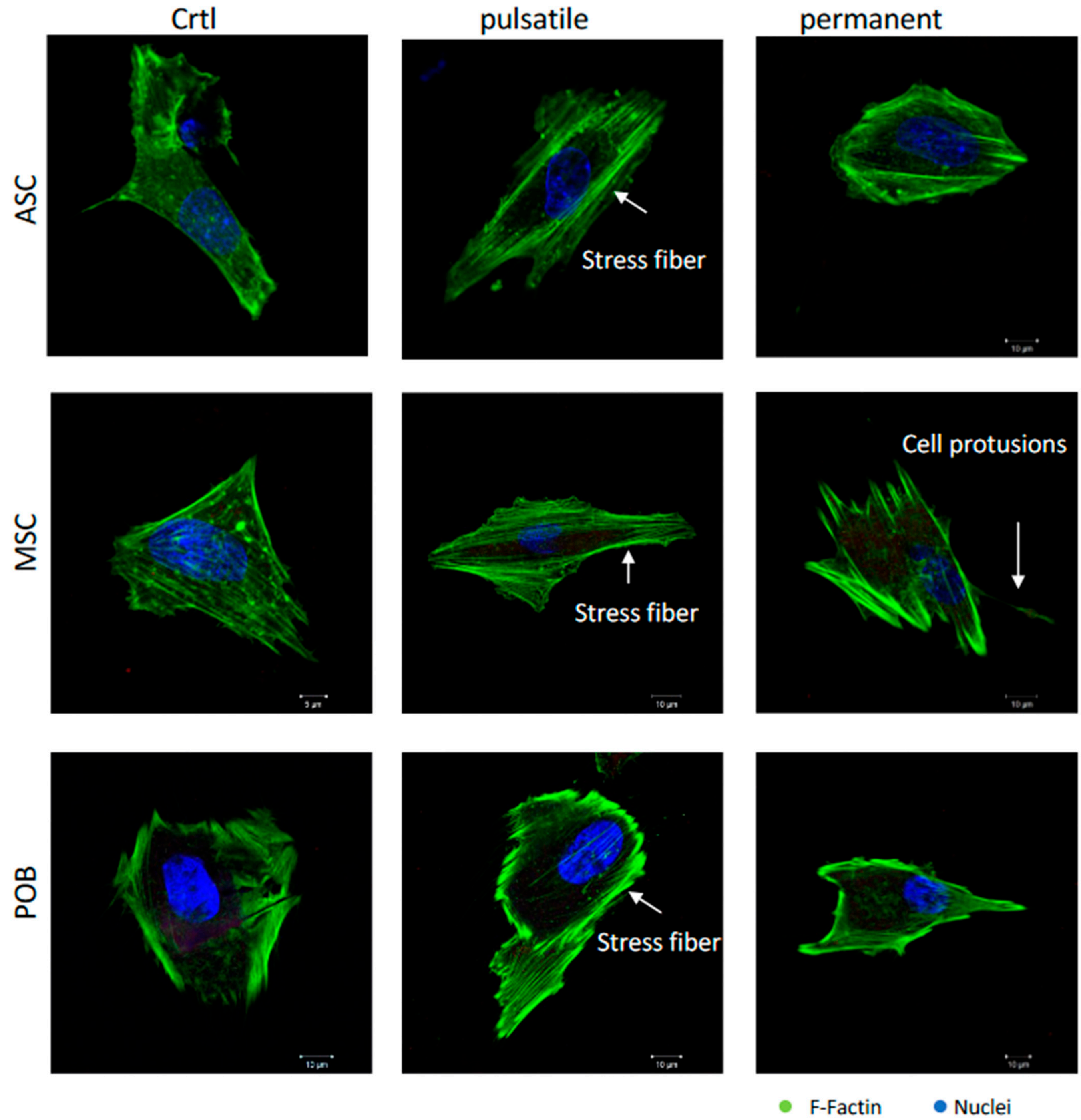

Figure S3. Alterations in F-actin structure.
